# Insomnia and depressive symptoms in relation to unhealthy eating behaviors in bariatric surgery candidates

**DOI:** 10.1186/s12888-018-1734-7

**Published:** 2018-05-29

**Authors:** Małgorzata Wrzosek, Marcin Wojnar, Ada Sawicka, Marek Tałałaj, Grażyna Nowicka

**Affiliations:** 1 Department of Biochemistry and Pharmacogenomics, and Center for Preclinical Studies, Medical University of Warsaw, Banacha 1, 02–097 Warsaw, Poland; 2 Department of Psychiatry, Medical University of Warsaw, Warsaw, Poland; 3 Department of Geriatrics, Internal Medicine and Metabolic Bone Diseases, Medical Centre of Postgraduate Education, Prof. W. Orlowski Hospital, Warsaw, Poland

**Keywords:** Obesity, Insomnia, Depression, Eating habits

## Abstract

**Background:**

Alongside obesity, insomnia and depression are common public health problems. Sleep problems are currently believed to be associated with excessive food intake and metabolic disturbances. Therefore, we aimed to explore a relationship between insomnia, depressive symptoms and eating habits as well as metabolic parameters in bariatric surgery candidates.

**Methods:**

A total of 361 unrelated obese subjects were included in this study. Severity of sleep problems was measured with Athens Insomnia Scale (AIS) and the severity of depressive symptoms was assessed with the Beck Depression Inventory (BDI-II). Obstructive sleep apnea (OSA) was assessed by the Apnea Hypopnoea Index (AHI). Information was obtained about demographics, eating habits and lifestyle. Blood samples were collected to measure concentration of lipids (cholesterol, triglyceride, HDL-cholesterol, LDL-cholesterol), and glucose.

**Results:**

The median (interquartile range) score for AIS in the study participants was 5 (3–8) with a range of 0–24 and 47% (171) participants scored ≥6 (met criteria for diagnosis of insomnia). Statistically significant correlations were found between the AIS scores and serum triglycerides and glucose concentrations, and BDI-II total scores. The highest scores on AIS and BDI-II were found in participants with high frequency of snack food consumption, in physically inactive individuals as well as in those who self-reported eating at night or who declared more than 3 intense emotions associated with a desire-to-eat. Adjusted multivariate logistic regression analysis revealed that clinical insomnia was most strongly associated with daily consumption of snack foods, with the odds ratio of 3.26 (95% CI: 1.74–6.11), while depressive symptoms were strongly associated with both eating in response to ≥3 specific emotions with OR = 2.93 (95% CI: 1.26–6.78) as well as with daily consumption of snack foods with OR = 2.87 (95% CI: 1.16–5.14).

**Conclusions:**

The results indicate that insomnia and depression in obese individuals are associated with eating habits, and suggest that in some patients these associations appears as major factors affecting obesity development.

## Background

Obesity, which reflects energy imbalance related to increased dietary intake and low energy expenditure, has become one of the world’s most significant health problems in recent few decades [1]. The effectiveness of obesity treatment based on reduction of food intake and increase in energy expenditure is low, both at population and patients’ levels [2]. It results in an increase in the number of morbidly obese patients for whom bariatric surgery is the only method leading to a significant weight loss. Many patients, however, fail to maintain the achieved weight and become morbidly obese again. Therefore, a better understanding of factors that impede obesity treatment is needed to develop comprehensive and effective therapy. Eating and sleeping are two kinds of behavior that are essential for the survival of humans [3]. Sleep is a major modulator of hormone release and glucose metabolism regulation. Food intake is controlled by the neuroendocrine system and the central nervous system [4]. Previous studies have demonstrated that short sleep duration as well as experimental sleep deprivation in healthy humans increase hunger and appetite [5, 6]. In a cross-sectional study in Japanese females poor sleep quality was found to be significantly associated with consumption of energy drinks and sugar-sweetened beverages, skipping breakfast, and eating irregularly [7]. This finding suggests that unhealthy food habits may be associated with insomnia symptoms, and prospective weight gain in individuals with sleep disorders. Thus, the crosstalk between sleep quality and metabolism plays a key role in the regulation of food intake and energy balance, affects obesity development and should be taken into account in both obesity treatment and prevention. Insomnia is the most common sleep disorder [8]. The insomnia diagnosis according to the fourth version of the Diagnostic and Statistical Manual of Mental Disorders (DSM-IV) includes difficulties in initiating or maintaining sleep or experiencing non-restorative sleep for a period of 1 month or more and the symptoms result in a significant impairment in daily functioning [9]. In clinical practice to measure insomnia symptoms Athens Insomnia Scale (AIS) is commonly used [10, 11]. Various risk factors for insomnia have been identified in the general population. Female gender and obesity were reported to increase the risk of chronic insomnia [12]. Previous studies have indicated that individuals with obesity are significantly more likely to report insomnia [13, 14], which could suggest that some individuals sleep worse because they are obese. This could be due to obstructive sleep apnea (OSA) that often coexists with obesity [15]. It was shown that among individuals presenting complaints related to sleep apnea, the co-occurrence of insomnia varies between 6 and 84% [16]. On the other hand, insomnia may predispose to overconsumption of energy or night eating, thus leading to weight gain [14]. Unhealthy behaviors, including unhealthy food choices and consuming excessive amounts of food after sleepless night, are probably driven by coping mechanisms and hedonic stimuli processing in the brain. Thus snacking unhealthy food is not performed to satisfy hunger or thirst, but to compensate for sleep disturbances and to improve mood [17]. There is an evidence for a combined relationship between unhealthy eating and depressive symptoms [18, 19]. Snacking on food and/or beverages was associated with an increased odds ratio for depression in 24,697 Japanese adults after adjusting for sleep problems [18]. While in a study in 376 Japanese adults, participants with more than two unhealthy eating behaviors had a higher incidence of depressive symptoms compared to those with fewer than two unhealthy eating behaviors [19]. Also nocturnal snacking was associated with greater depressive symptoms and it was shown that the nocturnal snacking was higher among the bariatric surgery candidates than among participants from general community [20].

Insomnia co-occurs with depression [21] and can have serious consequences [22]. Several recent studies have demonstrated that insomnia is associated with metabolic disorders. [23–26]. It was shown that both sleep disturbances (difficulty in initiating or maintaining sleep) and sleep duration (< 5 h or more than 9 h) are risk factors for type 2 diabetes [27, 28] and in patients with type 2 diabetes poor sleep quality as assessed by the Pittsburg Sleep Quality Index (PSQI) was associated with longer duration of diabetes [29, 30] and poor glycemic control [30]. On the other hand, insomnia symptoms were recognized to be associated with physical inactivity [31, 32]. Reduced motivation to exercise may be caused partly by tiredness. There is some evidence supporting physical activity as a nonpharmacologic treatment for sleep disturbance [33].

Improving sleep quality appears as an important point of intervention, which can enhance effectiveness of obesity therapy including effectiveness of bariatric surgery associated weight loss. The evidence of adverse effects of poor sleep on dietary intake is based mainly on studies of experimental reduction of sleep duration [34, 35], and there is a lack of studies in which objective measures of sleep were used. For the present study, we hypothesized that sleep problems as assessed by Athens Insomnia Scale (AIS) are associated with unhealthy eating habits in bariatric surgery candidates. Moreover, the relationships between the insomnia and depressive symptoms, sedentary lifestyle, and obstructive sleep apnea (OSA) that often coexists with obesity may be of importance for effective obesity treatment.

## Methods

### Participants

The current study comprises a group of patients who were recruited by clinical staff members at the time of a routine evaluation prior to bariatric surgery. Data were retrospectively collected based on clinical examinations between March 2013 and March 2016. All participants were Caucasian from European ancestry. Written informed consent was obtained from each participant after a full explanation of the study. The study protocol was approved by the Institutional Bioethics Committees (KB/127/2012 at the Medical University of Warsaw; 7/PB/2015 at the Medical Centre of Postgraduate Education). Criteria for exclusion from the study were as follows: use of antipsychotics or antidepressants, current pharmacotherapy for insomnia, acute endocrine dysfunction, chronic kidney disease, and alcohol use disorder.

### Procedures and assessment measures

Overnight fasting blood samples were taken from all participants. Standard assays were used to measure total cholesterol, high-density lipoprotein cholesterol, triglycerides, and glucose. Low-density lipoprotein-cholesterol levels were calculated using the Friedewald formula [36].

In all subjects, anthropometric measurements (body weight and height) were taken and body mass index (BMI) was calculated as the ratio of weight (kilograms) to the square of height (meters). Obstructive sleep apnea (OSA) was assessed by the Apnea Hypopnoea Index (AHI), which is the number of complete (apneas) or incomplete (hypopneas) obstructive events per hour of sleep. All participants underwent standard overnight assessment (polysomnography, PSG) to evaluate presence of OSA. OSA was defined as an Apnea Hypopnea Index of five or greater (AHI ≥ 5) [37]. The OSA group was divided into 3 severity stages: mild (5 ≤ AHI < 15), moderate (15 ≤ AHI ≤ 30), and severe (AHI > 30) [37]. All-night hemoglobin oxygen saturation (SpO2) was obtained with a finger oximeter. Polysomnographic variables were not valid to be used to evaluate insomnia [38].

Information on age, education, and a detailed clinical history, including history of obesity [39] was obtained for each patient, and a full physical examination was performed as a part of the preoperative evaluation process. The participants, under supervision of a multidisciplinary team, completed a battery of self-reported measures. At baseline all participants completed a questionnaire packet, including the Athens Insomnia Scale (AIS) and the Beck Depression Inventory (BDI-II). Details on Polish translation of these instruments were described elsewhere [40]. The AIS was used to determine the presence of clinical insomnia. The AIS is a validated, effective, self-assessment psychometric instrument designed for quantifying severity of sleep problems based on the ICD-10 criteria [10]. The AIS items measure awakenings during the night, early morning awakening, total sleep duration, sleep quality and sleepiness during the day. The scale has eight questions. Each question could be rated from zero (no problem) to three (very serious problem). AIS total score is the sum of the scores on each question and may vary from zero to 24. Scores of six or higher indicate the presence of insomnia (AIS sore ≥6). Depressive symptoms were measured using the Beck Depression Inventory (BDI-II) [41]. The BDI-II has 21 items rated on an intensity scale of 0–3 with a maximum score of 63, and its reliability and validity in mental health contexts are well established. A BDI-II score ≥ 14 is considered a positive screen for depressive symptoms [42].

A number of introductory questions designed to obtain general picture of participant’s eating habits, similar to questions of the Eating Disorders Examination-Questionnaire (EDE-Q), were used and we recognized how many times in the past month participants had had an episode of eating after they had gone to bed (i.e. night eating) [43]. We rated night eating frequency using a 0 to 3 scale: 0 = Not eaten; 1 = Eaten seldom, 2 = Eaten often, 3 = Eaten every day. Participants were also asked about snack-type foods and requested to circle snacks (energy-dense, nutrient-poor foods such as biscuits, cakes, sweets, chocolate, crisps, nuts, ice-cream) that they usually eat. Then, they rated the frequency of eating those snacks over the past month on the following 3-point scale: 0 = Not eaten, 1 = Eaten sometimes, 2 = Eaten every day. To measure the number of specific emotions associated with a desire-to-eat we used items developed with language similar to that used in the Emotional Overeating Questionnaire (EOQ) [44]; we focused on the most commonly experienced emotions according to the previous research [45, 46]. Studied individuals who reported eating in response to various emotional states, were asked to mark the type of emotions, choosing from: happy, lonely, depressed, bored, sad, angry, stressed, frightened, love, surprised, upset, and anxious [46].

Finally, variables in the questionnaire included a description of weekly aerobic physical activities [47]. All subjects were asked to self-defined their physical activity and answer a few questions to give better insight in working day and weekend physical activity. Patients described their physical activity (PA) using the following 4-point scale: 0 = Inactive, 1 = Low PA, 2 = Moderate PA, 3 = High PA, and were asked about amount and intensity of activities such as walking, jogging, cycling, swimming, gym, gymnastics, rehabilitation, fitness, dance, team games [47]. Patients physically inactive or with low physical activity reported also if they were aware of the importance of physical activity for health, and pointed to various reasons which, in their opinion, made them difficult to follow advices to enhance their activity.

### Data analysis

Statistical analysis was performed with the Statistica software package, version 12.0. The concordance with normal distribution for all variables was calculated with the Shapiro-Wilk and Kolmogorov-Smirnov tests. If the data were not normally distributed, we used nonparametric tests. Descriptive statistics were presented as medians and interquartile range (quartiles 1 and 3). Categorical variables were described with number (percentage). Correlations between variables and AIS scores were analyzed using Spearman’s correlation coefficient. Association tests were performed using Kruskal-Wallis tests and Mann-Whitney rank tests with the eating habits (unhealthy snack consumption, eating at night, and eating in response to specific emotions), physical activity and the AIS and BDI-II scores entered as dependent variables. Logistic regression was used to compute crude odds ratios (ORs) with 95% confidence intervals (CIs) for variables associated with insomnia (AIS score ≥ 6) or depression (BDI-II score ≥ 14). Variables that were significantly related with insomnia or depression in univariate analyses were then entered into the multivariate logistic regression model to estimate adjusted ORs with 95% CIs. In all analyses, a *p*-value < 0.05 was considered statistically significant.

## Results

Out of 436 severely obese individuals who agreed to participate in this study and provided informed consent, 361 subjects successfully completed the Athens Insomnia Scale (AIS) and Beck Depression Inventory (BDI-II), and returned all other questionnaires; thus a response rate was 83%.

### Patients characteristics

The mean age of the study group was 43.6 ± 11.5 years and the mean BMI was 42.3 ± 6.4 kg/m^2^. The majority of the participants (63%) had a BMI above 40 kg/m^2^, 28% had a BMI in the range of 35–39.9 kg/m^2^, and 9% of the subjects had class I obesity (BMI: 30.0–34.9 kg/m^2^). The study group had a significantly higher representation of women (73%) than men (27%; *p* < 0.001). About 28% of participants had a Master’s Degree, 19% had Bachelor’s Degree, 46% had some tertiary education, 1% had secondary education, and the remaining 6% had primary education.

### Insomnia and depression

The median (interquartile range) score for AIS in the study participants was 5 (3–8). Based on the AIS, insomnia was diagnosed in 47% (171) participants scoring ≥6 (higher cut-off score). No statistically significant correlation between the AIS scores and education level was found (Rho = 0.058, *p* = 0.293). No significant differences in the AIS scores between BMI-based categories of obesity were found (Kruskal-Wallis test: H = 0.535, *p* = 0.765). Likewise, no correlation was found between insomnia severity (AIS) and BMI (Table 1). Strong correlation was recognized between the AIS and BDI-II total scores (Table 1).

**Table 1:** Correlations between Athens Insomnia Scale (AIS) scores and clinical variables in patients with obesity

	Median (IQR)	Athens Insomnia Scale (AIS)	
		Rho	p
BDI-II	10 (5–16)	**0.576**	**0.000**
Age (years)	43 (35–53)	0.105	0.054
Weight (kg)	118 (103–133)	−0.061	0.262
Height (cm)	168 (162–174)	−0.107	0.050
BMI (kg/m2)	41 (37–45)	−0.021	0.698
Fat (%)	44 (39–47)	0.029	0.624
Glucose (mg/dl)	98 (90–115)	**0.115**	**0.035**
Total cholesterol (mg/dl)	183 (154–211)	0.011	0.836
LDL-cholesterol (mg/dl)	111 (84–133)	−0.074	0.184
HDL-cholesterol (mg/dl)	40 (34–47)	−0.002	0.964
Triglycerides (mg/dl)	139 (101–188)	**0.119**	**0.028**
Apnea Hypopnea Index (AHI)	5 (1–15)	−0.0167	0.772
Number of desaturations	24 (6–72)	−0.003	0.960
Average oxygen saturation (%)	94 (92–95)	−0.098	0.076
Minimum oxygen saturation (%)	86 (81–89)	−0.061	0.274

Spearman’s correlations, *p* values were considered significant when *p* > 0.05 (in bold). Data is presented as median, interquartile range (IQR)

*AIS* Athens Insomnia Scale, *BDI-II* Beck Depression Inventory, *BMI* body mass index, *HDL* high-density lipoprotein, *LDL* low-density lipoprotein

BDI-II scores analysis revealed that 3% (*n* = 11) of the study participants scored 29–63, which indicates severe depression, 14% (*n* = 52) scored 20–28 indicating moderate symptoms, 19% (*n* = 67) participants scored 14–19 corresponding to mild depression, and 64% (*n* = 231) scored 0–13 corresponding to minimal symptoms according to generally accepted BDI-II scores interpretation [42].

### Metabolic parameters and the prevalence of OSA

In the study participants, 29% were diagnosed with type 2 diabetes, and in 14% increased fasting glucose concentrations were found, indicating disturbances in glucose metabolism. In patients with T2D, 43% patients received hypoglycemic drugs, 32% patients received insulin, while 25% patients received insulin and hypoglycemic drugs. Patients with dyslipidemia who were taking hypolipidemic drugs received statins (74%), fibrates (16%) or statins plus fibrates (10%). Despite treatment, in 42% of the patients, total cholesterol was above 190 mg/dL; in 46%, LDL-cholesterol exceeded 115 mg/dL; and in 21%, triglycerides levels were above 200 mg/dL. Weak but statistically significant correlations were found between the AIS scores and triglycerides and glucose concentrations (Table 1).

The median (interquartile range) Apnea Hypopnea Index for the entire sample was 5 (1–15) with 50% participants with AHI ≥ 5, referred to as the OSA group [37]. PSG revealed that 25% (*n* = 90) patients had mild OSA (5 ≤ AHI < 15), 14% (*n* = 50) had moderate OSA (15 ≤ AHI ≤ 30), and 11% (*n* = 40) had severe OSA (AHI ≥ 30). No significant differences in the AIS scores between OSA group (AHI ≥ 5) and non-OSA group (AHI < 5) were found (Mann-Whitney U test, *p* = 0.850). In addition, no significant differences in the AIS scores between 3 stages in OSA group (mild, moderate and severe OSA) were found (Kruskal-Wallis test: H = 0.535, *p* = 0.765). Likewise, no correlation was observed between AIS scores and AHI (*p* = 0.772, Table 1). AIS did not correlate with number of desaturations or with average (Av SaO2) and minimum (Min SaO2) oxygen saturation (Table 1).

### Eating habits, sedentary lifestyle, insomnia and depressive symptoms

Sixty-six participants (18%) reported daily consumption of snack foods and they had the highest AIS and BDI-II scores (Table 2). Of the studied patients with obesity, 65% described their physical activity as low or reported no psychical activity and classified their lifestyle as sedentary. We found that individuals who reported “no”, “low” or “moderate” physical activity had significantly higher AIS sores than those with self-reported “high” physical activity (Table 2).

**Table 2:** Eating habits and physical activity in relation to the Athens Insomnia Scale and Beck Depression Inventory

	N	Athens Insomnia Scale (AIS)	Beck Depression Inventory (BDI-II)
Eating snack-type foods			
Every day	66	7 (5–10)	15 (10–23)
Occasionally	260	4 (3–8) ^**^	10 (5–15) ^***^
Not at all	35	4 (2–8) ^**^	8 (4–14) ^***^
*Statistics*		*p* = **0.0001**	*p* = **0.0000**
Number of emotions associated with desire to eat			
3 or more	29	9 (6–13) 6 (3–9)	19 (11–25) 11 (6–16)
< 3	214		
*Statistics*		*p* **= 0.0009**	*p* **= 0.0002**
Night eating			
Every night	7	9 (3–13)	19 (12–21)
Often	56	7 (4–11) ^ns^	10 (6–20) ^ns^
Rarely	96	6 (3–9) ^ns^	11 (7–16) ^ns^
Never	202	5 (3–7) ^*^	10 (5–15) ^*^
*Statistics*		*p* = **0.0009**	*p* = **0.0131**
Physical activity			
Inactive	50	7 (4–10) ^***^	14 (8–20) ^***^
Low	185	5 (4–8) ^***^	11 (6–16) ^*^
Moderate	116	4 (3–9) ^**^	9 (5–14) ^ns^
High	10	2 (1–3)	3 (0–8)
*Statistics*		*p* **= 0.0000**	*p* **= 0.0001**

Data are presented as median, interquartile range

Analysis was performed using Kruskal-Wallis tests and Mann-Whitney rank tests, where appropriate; p values were considered significant when p > 0.05 (in bold); Post-hoc analyses were conducted to determine differences in AIS and BDI-II scores between the groups and group eating snack-type foods every day, group eating every night, group with high physical activity

* *p* < 0.05, **p < 0.01, ***p < 0.001, ns – non-significant

Of the studied patients, 2% reported daily episodes of eating at night, which was associated with high AIS and BDI-II scores (Table 3). Moreover, 67% (*n* = 243) of studied individuals reported eating in response to various emotional states, and significantly higher AIS and BDI-II scores were found in this group in comparison to the subjects who did not report eating when emotional. In addition, participants who reported more emotions (3 or more) associated with a desire-to-eat had higher AIS and BDI-II scores than those who reported fewer emotions (see Table 2).

**Table 3:** Results of a logistic regression - crude and adjusted odds ratios for factors associated with insomnia and depression in bariatric surgery candidates

Variable	Athens Insomnia Scale sore ≥6				Beck Depression Inventory-II sore ≥14			
	Crude OR (95% CI)	p_a_	Adjusted OR (95% CI)	p_b_	Crude OR (95% CI)	p_a_	Adjusted OR (95% CI)	p_b_
Daily consumption of snack foods	**3.76 (1.58–8.97)**	**0.0022**	**3.26 (1.74–6.11)**	**0.0002**	**3.42 (1.96–5.96)**	**0.0000**	**2.87 (1.16–5.14)**	**0.0007**
Eating in response to ≥3 specific emotions	**3.56 (1.47–8.50)**	**0.0046**	**2.86 (1.14–5.13)**	**0.0235**	**3.53 (1.57–7.94)**	**0.0021**	**2.93 (1.26–6.78)**	**0.0117**
Night eating	**1.54 (1.01–2.35)**	**0.0434**	1.24 (0.79–1.93)	0.3472	**1.54 (0.99–2.38)**	**0.0416**	1.22 (0.76–1.94)	0.3989
Physical inactivity	**2.22 (1.19–4.12)**	**0.0104**	1.48 (0.75–2.90)	0.2492	**2.14 (1.17–3.93)**	**0.0131**	1.44 (0.75–2.79)	0.2699

*OR* odds ratio, *CI* Confidence interval, *p*-value < 0.05 are bolded

^a^Crude logistic regression analysis

^b^Adjusted logistic regression analysis (after controlling for all other significant factors)

In univariate analysis, we found that daily consumption of snack foods, self- reported eating in response to more than 3 emotions, night eating and physical inactivity were all significantly associated with clinical insomnia (AIS score ≥ 6) and depression (BDI-II score ≥ 14) (Table 3). Adjusted multivariate logistic regression analysis revealed that clinical insomnia was most strongly associated with daily consumption of snack foods, with the odds ratio of 3.26 (95% CI: 1.74–6.11), while depressive symptoms were strongly associated with both eating in response to ≥3 specific emotions with OR = 2.93 (95% CI: 1.26–6.78) as well as with daily consumption of snack foods with OR = 2.87 (95% CI: 1.16–5.14) (Table 3). Additionally, in adjusted analysis insomnia was the strongest predictor of daily consumption of snack foods (Table 4); this association was even stronger than for depression. This indicates a bidirectional relation between the daily consumption of snack foods and insomnia.

**Table 4:** Results of the logistic regression - crude and adjusted odds ratios for factors significantly associated with a daily consumption of snack foods in bariatric surgery candidates

Variable	Daily consumption of snack foods			
	Crude OR (95% CI)	p_a_	Adjusted OR (95% CI)	p_b_
Athens Insomnia Scale sore ≥6	**3.95 (2.16–7.20)**	**0.0000**	**3.06 (1.56–5.86)**	**0.0007**
Beck Depression Inventory-II sore ≥14	**2.56 (1.47–4.47)**	**0.0000**	**2.11 (1.14–3.93)**	**0.0168**
Eating in response to ≥3 specific emotions	**2.78 (1.21–6.33)**	**0.0153**	1.83 (0.77–4.37)	0.1693

*OR* odds ratio, *CI* Confidence interval, *p*-value < 0.05 are bolded

^a^Crude logistic regression analysis

^b^Adjusted logistic regression analysis (after controlling for all other significant factors)

## Discussion

The present study shows that the participants reporting daily consumption of snack foods and eating in response to 3 or more emotions had the highest AIS and BDI-II scores. Unhealthy eating may be a coping mechanism for sleep deficit as well hard digestible food may deteriorate the quality of sleep. Therefore, the bidirectional relation between sleep disturbances and eating habits can be suggested. Nevertheless, it is not clear from our cross-sectional study whether sleep disturbances influence consumption of snack foods or vice versa. Very few previous findings suggest that unhealthy food habits are associated with insomnia symptoms. The relationship between dietary intake and sleep was examined in Japanese female workers. The results showed that low intake of vegetables, high intake of confectionary, and unhealthy eating habits were associated with poor sleep quality assessed using the Pittsburgh Sleep Quality Index (PSQI) [7]. On the contrary, mainly weak and inconsistent associations between insomnia symptoms and poor food habits in Helsinki Health Study were found [31]. However, the Finnish study was unable to confirm any associations probably due to general questions on food habits and suggestive categorization of food habits as healthy or unhealthy. On the other hand, an experimental reduction of sleep duration was accompanied by increased intake of calories from snacks [34, 48], increased food purchasing in normal-weight men [35], and increased hunger and appetite, especially for calorie-dense foods with high carbohydrate content [5]. These associations seem to be related to the alterations in appetitive brain signaling [49]. Neural activation was measured by functional magnetic resonance imaging (fMRI) in twenty-three healthy participants examined on two sessions: a night of normal sleep and a night of total sleep deprivation. Sleep deprivation significantly decreased activity in the anterior cingulate cortex, lateral orbital frontal cortex and anterior insular cortex (brain regions known to be instrumental in appetitive desire and food stimulus evaluation) and increased the amygdala responsivity to desirable food items. Additionally, increase in desire for high-calorie foods, which positively correlated with the severity of sleep deprivation, was demonstrated [49]. Thus, deeper insight in potential determinants of eating behaviors is needed to identify new effective strategies for obesity prevention and treatment. The prevalence of insomnia in our study (47% participants scoring ≥6 on the AIS) is similar to insomnia frequency assessed in the population of 6079 Latin American women aged 40 to 59 years (43.6% had insomnia, AIS) [50]. Also sedentary lifestyle, defined as fewer than three weekly 30-min periods of physical activity, was very common in surveyed American women (63.9% of them were self-defined as sedentary). Sedentary women had more depressive and insomnia symptoms as compared with non-sedentary women. As expected, in our report 65% studied patients with obesity described their physical activity as low or declared psychical inactivity and classified their lifestyle as sedentary. The statistical analysis revealed that these individuals had significantly higher AIS and BDI-II scores than those with self-reported “high” physical activity. It has been considered that cultivating exercise habits, reducing sedentary time and improving sleep quality may be important strategies for obesity prevention [51–53]. Our present research indicates that insomnia and depression symptoms commonly coexist in obese individuals, and it is consistent with findings of a meta-analysis of 19 original papers reporting that sleep deprivation alters mood [54] and the results of a large prospective population-based study — the Nord-Trøndelag Health Studies (HUNT2 and HUNT3) showing that insomnia is associated with more than twice the odds for depression [22].

Sleep disturbances are considered as a risk factor of obesity [55, 56]. However, available findings are inconsistent and high BMI was reported to be not associated with the insomnia symptoms or insomnia as a syndrome [57, 58]. We also found no correlation between BMI and Athens Insomnia Scale scores. Nonetheless, it should be emphasized that participants in our study had extremely high BMI. In addition, insomnia might be directly associated with increased consumption of palatable food, but not with a general tendency to eat more. This suggests that insomnia symptoms may contribute to unhealthy eating patterns or increased amounts of energy consumed at inappropriate time points, i.e., at night. It is important to note that other studies revealed late-night eating in response to sleep restriction [59], insomnia [14] or depressive symptoms [20]. Similarly, 2% of the studied bariatric surgery candidates reported daily episodes of eating at night, which was associated with the highest AIS scores (median = 9) and BDI-II scores (median = 19). However, interpretation of our findings is limited due to the small group of every night eating individuals.

In the present study, the AIS and BDI-II scores were high in participants who self-reported eating in response to more than 3 different emotions. Emotions may affect human eating behavior. Indeed, in the current study we demonstrated that depression was associated with a tendency to eat in response to more than 3 emotions as well as with unhealthy snack consumption among individuals with obesity. Our data support the previous findings showing that emotional eating can be associated with increased BMI [60] and with depressive symptoms [61].

Adequate sleep quality and duration are important for the appetite regulation and normal functioning of metabolic and hormonal processes [62]. Moreover, many experimental and epidemiologic studies link sleep disturbance with alterations in glucose homeostasis [63]. For instance, it was shown that short sleep time (4 h per night for 6 nights) in young, healthy men was associated with lower glucose tolerance than in the fully rested men [64]. In healthy individuals, glucose tolerance varies throughout the day; glucose concentrations are markedly higher in the evening than in the morning, and glucose tolerance is reduced in the night [65]. Circadian rhythm is important modulator of glucose homeostasis and sleep loss may result in metabolic alterations. In our study, high AIS scores were associated with high glucose and triglycerides concentrations in bariatric surgery candidates. This finding is consistent with the hypothesis that changes in the quantity or quality of sleep may affect carbohydrate and lipid metabolism. The amount of research investigating the relationship between insomnia symptoms and glucose and lipid abnormalities in obese patients is limited. However, prior reports demonstrated that shift workers from two plants of northern France had significantly higher levels of serum triglyceride than day workers, but there was no influence of shift work on total cholesterol and HDL cholesterol concentrations [66].

Obstructive sleep apnea (OSA) is a highly prevalent respiratory disorder, characterized by recurrent episodes of upper airway obstruction occurring during sleep, and associated with recurrent cycles of desaturation and re-oxygenation [67]. Although OSA is characterized by fragmented sleep, not all affected patients complain of insomnia [12, 16, 68]. In our study, no correlation between AHI and insomnia symptoms as assessed with AIS was observed, however, 25% of the study participants had mild OSA and only 11% had severe OSA. Our results support the hypothesis that patients with OSA of mild to moderate severity may not necessarily report low quality of sleep, as it was previously suggested [69].

The current findings should be considered in the context of several limitations. First, to assess insomnia we used the Athens Insomnia Scale, a self-report validated questionnaire, which is a subjective instrument to evaluate sleep disturbances. Unfortunately, we were not able to utilize PSG to measure objective electrophysiological parameters of sleep quantity and quality. Second, this study did not allow assessing insomnia symptoms in a relationship to amount of calories consumed, because we did not assess the overall caloric intake for each participant. We used self-report measurements of physical activity and there might be a concern that the recall of physical activity may be subjective in some cases. Our study also did not assess all the aspects of night eating, which may be relevant when diagnosing night eating syndrome (NES), i.e., consuming ≥25% of total daily calories after the evening meal, or the type of food consumed [70]. Instead, we have assessed night eating frequency using the Eating Disorder Examination Questionnaire (EDE-Q) [43]. Another limitation of our study may be related to a larger number of females than males. Therefore, future studies should focus upon identifying any potential gender differences. Our study participants had extremely high BMI and obesity-associated metabolic abnormalities, and some of them were under pharmacological treatment for dyslipidemia and/or type 2 diabetes. Despite taking medications that improve lipid or glucose profile we still could demonstrate in our patients the influence of sleep disturbances on studied biochemical parameters.

The dramatic increase in obesity prevalence overlaps the reduced sleep duration and quality, which are hallmarks of the modern society. Sleep disturbances and depressive symptoms may be considered as a risk factor, which contributes to unhealthy eating behaviors that might result in the development of obesity.

## Conclusions

The results of the study show new data on the associations between insomnia, depression and eating behaviors in bariatric surgery candidates, and highlight the importance of the potential consequences of poor sleep or depression in obesity. This should stimulate further research in this area leading to the development of innovative and effective strategies for obesity prevention and treatment.

## Abbreviations

AHI: Apnea Hypopnoea Index
AIS: Athens Insomnia Scale
BDI-II: Beck Depression Inventory
BMI: Body Mass Index
CIs: Confidence intervals
EDE-Q: Eating Disorders Examination-Questionnaire
EOQ: Emotional Overeating Questionnaire
fMRI: functional magnetic resonance imaging
HDL-C: High-density lipoprotein cholesterol
LDL-C: low-density lipoprotein-cholesterol
OR: Odds Ratio
OSA: Obstructive sleep apnea
PA: Physical activity
PSG: Polysomnography
